# Functional characterization and in vitro pharmacological rescue of KCNQ2 pore mutations associated with epileptic encephalopathy

**DOI:** 10.1038/s41401-023-01073-y

**Published:** 2023-03-17

**Authors:** Gui-mei Yang, Fu-yun Tian, Yan-wen Shen, Chuan-yan Yang, Hui Yuan, Ping Li, Zhao-bing Gao

**Affiliations:** 1 School of Pharmacy, Zunyi Medical University, Zunyi, 563000 China; 2 Zhongshan Institute for Drug Discovery, Shanghai Institute of Materia Medica, Chinese Academy of Sciences, Zhongshan, 528400 China; 3 Center for Neurological and Psychiatric Research and Drug Discovery, Shanghai Institute of Materia Medica, Chinese Academy of Sciences, Shanghai, 201203 China; 4 Department of Pediatrics, The First Medical Center of PLA General Hospital, Beijing, 100853 China; 5 Department of Pediatric neurology, Children’s Hospital of Fudan university at Xiamen, Xiamen, 361006 China; 6 School of Basic Medicine and Clinical Pharmacy, China Pharmaceutical University, Nanjing, 211198 China; 7 School of Pharmaceutical Sciences, Southern Medical University, Guangzhou, 510515 China

**Keywords:** developmental and epileptic encephalopathy (DEE), KCNQ2 pore mutations, retigabine (RTG), pynegabine (HN37), XEN1101, electrophysiology

## Abstract

Mutations in the KCNQ2 gene encoding K_V_7.2 subunit that mediates neuronal M-current cause a severe form of developmental and epileptic encephalopathy (DEE). Electrophysiological evaluation of KCNQ2 mutations has been proved clinically useful in improving outcome prediction and choosing rational anti-seizure medications (ASMs). In this study we described the clinical characteristics, electrophysiological phenotypes and the in vitro response to KCNQ openers of five KCNQ2 pore mutations (V250A, N258Y, H260P, A265T and G290S) from seven patients diagnosed with KCNQ2-DEE. The KCNQ2 variants were transfected into Chinese hamster ovary (CHO) cells alone, in combination with KCNQ3 (1:1) or with wild-type KCNQ2 (KCNQ2-WT) and KCNQ3 in a ratio of 1:1:2, respectively. Their expression and electrophysiological function were assessed. When transfected alone or in combination with KCNQ3, none of these mutations affected the membrane expression of KCNQ2, but most failed to induce a potassium current except A265T, in which trace currents were observed when co-transfected with KCNQ3. When co-expressed with KCNQ2-WT and KCNQ3 (1:1:2), the currents at 0 mV of these mutations were decreased by 30%-70% compared to the KCNQ2/3 channel, which could be significantly rescued by applying KCNQ openers including the approved antiepileptic drug retigabine (RTG, 10 μM), as well as two candidates subjected to clinical trials, pynegabine (HN37, 1 μM) and XEN1101 (1 μM). These newly identified pathologic variants enrich the KCNQ2-DEE mutation hotspots in the pore-forming domain. This electrophysiological study provides a rational basis for personalized therapy with KCNQ openers in DEE patients carrying loss-of-function (LOF) mutations in KCNQ2.

## Introduction

The M-current is a voltage-gated potassium current characterized by subthreshold activation, slow activation kinetics, and lack of inactivation, involved in regulating neuronal excitability by limiting repetitive firing during long-lasting depolarizing inputs [1, 2]. KCNQs encoding for K_V_7 subunits are the molecular entity mediating M-current [3], and the K_V_7.2 subunit encoded by the KCNQ2 predominantly contributes to the molecular composition, forming heteromeric channels with K_V_7.3 (KCNQ3) throughout the central nervous system [4, 5]. Due to the prominent role in maintaining physiological neuronal excitability, pathogenic variants in KCNQ2 account for a wide spectrum of early-onset human epileptic disorders, ranging from self-limited familial neonatal epilepsy (SeLFNE; OMIM: 121200) to developmental and epileptic encephalopathy (DEE7, KCNQ2-DEE; OMIM: 613720) [6–9]. Most SeLFNE patients typically present seizures following a benign course with spontaneous remission and good neurodevelopmental outcomes [10, 11], while patients with KCNQ2-DEE face more severe conditions. The primary clinical features of KCNQ2-DEE include early-onset prominent and intractable tonic seizures with the burst-suppression pattern or multifocal epileptic activity on electroencephalography (EEG), frontal lobe hypoplasia and basal ganglia or thalamus hyperintensities on MRI in some cases, and moderate to severe developmental impairment, presenting with severe intellectual disability, language delay, stereotypical behaviors, autistic-like features, and sleep disorder [9, 12–15]. Moreover, the developmental problems of many KCNQ2-DEE patients were not resolved even after controlling their seizures in early life, indicating independent effects of KCNQ2 variants on developmental consequences from seizures of KCNQ2-DEE, which traditional antiseizure drugs could not solve.

Functional KCNQ channels are formed by four subunits that together form a central pore, and each has six transmembrane segments (S1–S6) composed of a voltage-sensing domain (VSD; S1–S4), a pore-forming domain (S5–S6), an N-terminal region, and a long C-terminal region [16, 17]. Since hundreds of disease-causing mutations in the KCNQ2 have been identified, the possible genotype-phenotype correlations have been interpreted by multiple groups from diverse perspectives [18–22], which helped to translate findings from human genetics to effective therapies [8, 9]. Pathogenic variants in KCNQ2-DEE are nearly always missense resulting from single nucleotide substitutions, which clustered in four hotspots known to be vital for channel activity, including the S4 voltage-sensor, the pore, and two intracellular C-terminal calmodulin-binding helices [23], affecting the KCNQ2-forming channel function through multiple pathways including not only the conductance and voltage dependence but also the co-translational folding, subcellular localization and the protein-protein interaction [24–26]. However, the epileptic phenotypes and neurodevelopmental outcomes of KCNQ2-DEE might be primarily associated with the functional consequence of the mutations on M-current in most situations [8, 20], and mutations falling within different KCNQ2 gene positions can cause distinct functional consequences and further contributing to disease phenotypes.

In this study, we described the clinical characteristics of seven patients diagnosed with KCNQ2-DEE and reported the functional consequences of the five KCNQ2 mutations causing DEE to determine whether these mutations residing in the pore domain led to a similar electrophysiological abnormality. Furthermore, we evaluated the effects of KCNQ openers in clinical trials, including retigabine (RTG, trial number: NCT04639310 and NCT04912856), XEN1101 (trial number: NCT03796962, NCT05614063, and NCT05667142), and pynegabine (HN37, trial number: CTR20201676 and CTR20222616), on the mutant channels to assess their potential in treating these children with KCNQ2-DEE.

## Materials and methods

### Patients

Seven DEE patients with pore mutations of KCNQ2 were recruited from the Chinese PLA General Hospital, Children’s Hospital of Fudan university at Xiamen, and consulting room studio at Hao Daifu Internet Hospital. Clinical data were collected, and the results of electroencephalography (EEG) were reviewed by a senior pediatric neurologist. Developmental and epileptic encephalopathy was diagnosed and classified according to the Commission on Classification and Terminology of the International League Against Epilepsy (ILAE) [27, 28]. The protocol for obtaining phenotype information on patients was approved by the ethics committee of the Chinese PLA General Hospital, and informed consent was obtained from the parents of the patients.

### Mutagenesis

The cDNA constructs for human KCNQ2 (NM_172107.4) and KCNQ3 (NM_004519.4) channels were subcloned into vector pcDNA5/FRT/TO or pcDNA3.1, respectively. The point mutations were introduced into the KCNQ2 cDNA using the QuikChange II kit (Agilent Technologies, CA, USA), and verified by fully sequencing (BGI Tech Solutions Co., Beijing, China).

### Cell culture and transient transfection

Chinese hamster ovary cells (CHO) were cultured in DMEM/F12 (Gibco, CA, USA) supplemented with 10% fetal bovine serum (Gibco, CA, USA) at 37 °C in a 5% CO_2_ incubator. For Western blotting and patch-clamp experiments, cells were seeded in 35-mm dishes and transfected the next day with appropriate plasmids using Lipofectamine 2000 reagent (Invitrogen, CA, USA). The plasmid of enhanced green fluorescent protein (EGFP) was used as a transfection marker (400 ng), and total plasmids in the transfection mixture were kept constant at 4000 ng. The amount of transfected wild-type KCNQ2 (KCNQ2-WT) or mutant plasmid was 3600 μg per well for the expression of homomeric channels; the amount of transfected KCNQ2-WT or mutant plasmid was 1800 ng per well with KCNQ3 plasmid of 1800 ng to mimic the expression of the heteromeric channel in homozygotes; the amount of transfected KCNQ2-WT, KCNQ2-mutant, and KCNQ3 were 900 ng, 900 ng, and 1800 ng to mimic the situation of patients (heterozygotes). A similar transfection configuration (2000 ng KCNQ2-WT/mutant + 2000 ng KCNQ3) was applied for Western blotting but without EGFP.

### Membrane protein extraction and Western blotting

Membrane and cytoplasmic proteins were extracted from CHO cells using the Mem-PER Plus Kit reagent (Thermo Scientific, MA, USA). Protein concentration was measured by BCA Protein Assay Kit (EpiZyme Biotechnology, Shanghai, China). Equal volumes and amounts of proteins were loaded with 15 well 10% SDS-PAGE gels (EpiZyme Biotechnology, Shanghai, China) and transferred onto nitrocellulose membranes using wet transfer at 200 mA/4 °C for 2 h. Membranes were blocked in blocking buffer (5% BSA dissolution in TBST) for 2 h and incubated with antibodies (Cell Signaling, MA, USA) against KCNQ2 (1:1000), β-actin (1:4000), or Na-K ATPase (1:1000) at 4 °C overnight. The nitrocellulose membranes were washed with TBST three times (15 min each) and incubated with anti-mouse IgG (1:10000) or anti-rabbit IgG (1:10000) secondary antibodies for 2 h. After being washed with TBST three times (15 min each), reactive bands were detected by enhanced chemiluminescence (ECL, Monad, Wuhan, China) using autoradiography films. Western blot band quantifications were analyzed using the ImageJ software.

### Whole-cell electrophysiology

Twenty-four hours after transfection, a whole-cell recording was performed at room temperature using an Axoclamp 700B amplifier and a Digidata 1550B (Molecular Devices, CA, USA). When filled with pipette solution, the resistances of glass micropipettes were 3.0 to 5.0 MΩ. The extracellular solution contained the following (in mM): 145 NaCl, 5 KCl, 2 CaCl_2_, 1 MgCl_2_, 10 HEPES, and 10 Glucose, adjusted pH 7.4 with NaOH; the pipette solution contained the following (in mM): 145 KCl, 1 MgCl_2_, 5 EGTA, 10 HEPES, and 5 ATP-Mg, adjusted pH 7.4 with KOH. The membrane potentials were held at −90 mV, then depolarized for 2 s from −90 to +60 mV with a 10-mV increment. The tail current elicited at −120 mV was measured to obtain a conductance-voltage (*G*-*V*) curve. Pynegabine (HN37), XEN1101, and retigabine (RTG) were provided by Dr. Fa-jun Nan (National Center for Drug Screening, Chinese Academy of Sciences, Shanghai, China). ICA-069673﻿ was purchased from MCE (MedChemExpress, Shanghai, China).

### Data analysis

Patch clamp data were processed using Clampfit 11.2 (Molecular Devices, CA, USA) and then analyzed in GraphPad Prism 8 (GraphPad Software). Voltage-dependent activation curves were fitted using the Boltzmann equation, *G* = *G*_min_ + (*G*_max_ – *G*_min_) / (1 + exp (*V* − *V*_1/2_)/*S*), where *G*_max_ is the maximum conductance, *G*_min_ is the minimum conductance, *V*_1/2_ is the voltage for reaching 50% of maximum conductance, and *S* is the slope factor. Activation rates were measured in Clampfit using a single exponential function: *I* = Ae^−t/τ^ + *C*. Current density (expressed in pA/pF) was calculated as peak K^+^ currents divided by cell capacitance. The data are presented as the mean ± SEM.

## Results

### The five identified KCNQ2 mutations are associated with severe clinical phenotype

The five KCNQ2 pore mutations (V250A, N258Y, H260P, A265T, and G290S) were from seven patients diagnosed with KCNQ2-DEE (Table 1). Of the seven patients, three were male, and four were female; all had seizure onset within the first 72 h of their lives, and only two had not reached seizure-free yet (patients 3 and 4). All patients had severe mental retardation except for two milder cases with the same variants of p.A265T (patients 5 and 6). Seizure-onset in these patients mostly contained the components of tonic seizures except for patient 5. The seizures in the neonatal period were most commonly accompanied by cyanosis; head deviation was also commonly seen. Three patients (patients 1, 3, and 4) had developed infantile spasms; all were at around three months old, much earlier than the peak onset age of most infantile spasms at about 6-months old. The patients who had developed infantile spasms also had delayed seizure control or could not reach seizure-free. Moreover, 85.7% (6/7) patients displayed a burst-suppression pattern EEG mainly during the neonatal period. Multifocal epileptic activity and delayed EEG background were the most reported interictal EEG (Supplementary Fig. S1). Hypsarrhythmia EEG could appear in patients who did not develop infantile spasms. Patients with a lower frequency of seizures during the neonatal period had better developmental outcomes (patients 5 and 6). Patients 5 and 6 also had Rett syndrome-like phenotypes; patient 6 also demonstrated autism-like behavior. Even though sound sensitivity appeared in three patients (patients 3, 4, and 7), startle-like non-epileptic myoclonus was more commonly seen (5/7). Patients 1, 3, 6, and 7 had additional sleep disorders, while patients 3 and 4 had nystagmus. Even though cranial magnetic resonance images were normal in most previously reported cases, slightly higher T1 signals on globus pallidus could be found usually during the neonatal period; some might also show slightly delayed myelination. Due to a delayed genetic diagnosis, phenobarbital was usually the first drug they received (6/7). All patients received combined therapy during some period; only one patient did not respond to oxcarbazepine.

**Table 1:** Clinical characteristics of KCNQ2-related encephalopathy patients.

Patient ID	1	2	3	4	5	6	7
Birthday	2018/11/16	2018/3/7	2020/11/26	2020/8/10	2020/9/16	2019/6/6	2020/1/26
Gender	Male	Male	Female	Male	Female	Female	Female
Genotype	c.749 T > C, p.V250A	c.749 T > C, p.V250A	c.772 A > T, p.N258Y	c.779 A > C, p.H260P	c.793 G > A, p.A265T	c.793 G > A, p.A265T	c.868 G > A, p.G290S
Onset-age of seizure	Within 24 h	4 h	Within 24 h (undetermined); 6 day (determined)	Within 24 h (undetermined); 3 day (determined)	Within 24–48 h	Within 24–48 h	Within 1 h
Origin of mutation	De novo	De novo	De novo	De novo	De novo	De novo	De novo
Seizure at onset	Tonic sz	Tonic sz; Tonic-clonic sz	Tonic sz with head deviation and cyanosis	Tonic sz with cyanosis; Tonic sz with opisthotonos and perioral cyanosis	Focal onset seizure (Blinking the left eye)	Tonic seizure with crying	Tonic sz with head deviation and cyanosis
Other seizure	Tonic-clonic sz; Tonic sz with cyanosis	Tonic-clonic sz; Tonic sz with cyanosis	Tonic sz with opisthotonos; Sticking out the tongue and spasm; Sticking out the tongue and tonic with spasm cyanosis; Grasp of hands especially before sleep	Tonic sz with head deviation, blinking eyes, spasm, grasp of hands	Tonic seizure with head deviation, lifted limbs and crying; Tonic seizures with cyanosis;	Tonic seizure with cyanosis; Tonic Seizures;14 m	Tonic-clonic sz
Frequency of seizures at most	100+/day	30+/day	30–40/day	40+/day	3/day	3–4/day	50+/day
Age of Seizure offset	18th month	20–30th day	No	No	42nd day	50–60th day	3rd month
Mental retardation	Yes: severe; can recognize the direction of sound from around 1 year; (Gesell3y3m: DQ6)	Yes: severe; (4 month 4 day: DQ3; Bayley 7 month 17 day: MDI35, PDI11) can walk with support around 4 year	Yes: severe; could not recognize the sound and image nor lift the head (16 month)	Yes: severe;1 year 7 month: smile, can recognize the direction of sound from around 6 month	Yes: mild; can recognize the direction of sound from around 3 month, can grasp with both hands at 7 month and turn around at 8 month, sit at 1 year 3 month, stand with assistant 1 year 6 month; pronounce papa at 1 year	Yes: mild; can recognize the direction of sound from around 3 month, sit at 6.5 month, walk with assistance at 14 month, walk by oneself at 18 month; cannot run or go up and down stairs alone	Yes: severe (3 month: DQ33) can recognize the direction of sound from around 6 month; 2 year 2 month: sit for moment
Sleeping disorder	Yes: from 2 year old	No	Yes: from 3 month old	No	No	Yes	Yes: from birth-3 month old; from 8 month old till now
EEG at onset	Multifocal epileptic activity with sleeping burst-suppression	Multifocal epileptic Activity	Delayed aEEG, equal to about 36 weeks without epileptic activity (5 day)	Multifocal epileptic activity	Burst-suppression	Immature background with intermittent burst-suppression and sporadic multifocal sharps	Multifocal epileptic activity mainly on left hemisphere
Burst-suppression pattern EEG	Yes: 10th day (during sleep)	No	Yes: 20th day (burst 2 s; suppress 3–5 s)	Yes: 1 month old (burst 2 s; suppress 2–5 s)	Yes: 20th day	Yes: 11th day	Yes: 1st day
Hypsarrhythmia EEG	No	No	Yes: atypical (4 month 16 day old)	Yes	No	Yes	No
Other EEG	Multifocal epileptic activity	Multifocal epileptic activity with slow-background rhythm	Multifocal epileptic activity and poor background	Multifocal epileptic activity with undeveloped occipital rhythm and general slow-wave	Multifocal sharps activity; slow-background with sporadic sharps	Multifocal sharps mainly on forehead; increased δ waves of background and multifocal epileptic activity; sporadic sharps on central area during sleep; normal; epileptic activity mainly on left side during sleep	Slow-waves with slightly slow-background rhythm and multifocal sleeping epileptic activity
EEG at CS	General suppression ended with sporadic sharps mainly at left hemisphere	None	Multifocal epileptic activity originated from frontal lopes; slow wave and suppressed amplitude followed by fast rhythm and sporadic sharps with increasing amplitude	Multifocal epileptic activity originated from hemisphere or generally onset and then followed by general slow-waves	General suppression followed by right frontal sharp-slow complex consisted for 1 min and then suppressed;	None	Multifocal epileptic activity
Cranial MRI	Slightly higher T1 on bilateral globus pallidus (17 days)	Normal	Normal	Right temporal lobe arachnoid cyst (1 month); slightly thin corpus callosum (4 monthes)	Slightly higher T1 on bilateral globus pallidus (20 days)	Slightly higher T1 on bilateral globus pallidus (6 day); normal (21 months)	Slightly high T1 on left globus pallidus (7 days);Slightly delayed myelination (18 months)
ASMs	PB, LEV, TPM, OXC, VGB, VPA	PB, midazolam, VitB6, CBZ, VPA, OXC	LEV, OXC, CZP, VGB	PB, LEV, VitB6, TPM, OXC, VPA, ZNS, CZP, Lacosamide	PB, VitB6, LEV, ACTH, VPA, OXC	PB, VitB6, TPM, LEV, OXC	PB, TPM, VitB6, VPA, LEV, OXC
Current ASMs	Ceased after 23 months	VPA, OXC	OXC, CZP	OXC, CZP, Lacosamide	OXC	OXC	OXC
ASMs with recommendation (R) or not recommendation (NR)	R: VGB; NR: OXC	R: VPA, OXC; NR: none	R: CZP; NR: none	R: OXC, CZP, Lacosamide; NR: none	R: OXC NR: LEV	R: OXC NR: none	R: OXC; NR: LEV

*SZ* seizure, *ASM* anti-seizure medications, *h* hour, *CS* clinical seizures, *ES* asymptomatic electrographic seizures, *EA* asymptomatic, epileptiform activity, *Caesarean Section* C-section, *PB* phenobarbital, *VitB6* Vitamin B-6, *LEV* levetiracetam, *TPM* topiramate, *OXC* oxcarbazepine, *VPA* valproic acid, *VGB* Vigabatrin, *ZNS* zonisamide, *CZP* clonazepam, *CBZ* Carbamazepine, *NA* not available.

### Functional properties of homomeric KCNQ2 channels carrying pore mutation

**Fig. 1:**
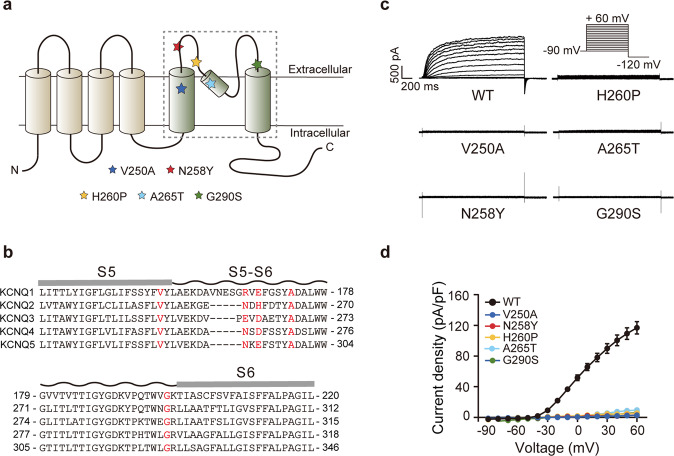
Functional characterization of the KCNQ2-WT, V250A, N258Y, H260P, A265T, and G290S homomeric channels. **a** Topology of KCNQ2 channel. Showing the six transmembrane segments, N-terminal domain, and C-terminal domain. The dashed-line boxes indicate the pore-forming domain; pentagrams highlighted in different colors indicate the location of the investigated mutations. **b** Sequence alignment within the pore-forming domain of human KCNQ1–5 channels. The five mutant residues are highlighted in red. **c** Representative current traces and **d** averaged current density-voltage relationship from cells expressing the KCNQ2-WT and mutant channels.

As shown in Fig. 1a, all five mutations, V250A, N258Y, H260P, A265T, and G290S, were located in the pore domain of the K_V_7.2 subunit consisting of the transmembrane segment S5, S6, and the S5–S6 linker between them. Based on the amino acid sequence alignment of the KCNQ family members, the four residues, V250, N258, A265, and G290, were highly conserved among KCNQ channels, while the amino acid at the position 260 in KCNQ2 was variable among the KCNQ family (Fig. 1b).

Using whole-cell patch-clamp recordings, we assessed the electrophysiological properties of these KCNQ2 mutations expressed in Chinese hamster ovary (CHO) cells. Wild-Type KCNQ2 channel (KCNQ2-WT) generated a slowly activating, non-inactivating, and outward current in response to depolarizing voltage steps from −90 to +60 mV when expressed alone; the macroscopic K^+^ current density at 0 mV was 51.6 ± 3.7 pA/pF, and the voltage of half activation (*V*_1/2_) was −7.1 ± 0.8 mV. Notably, cells expressing the five variants alone did not give detectable voltage-activated currents (Fig. 1c, d), suggesting loss-of-function (LOF) effects in these mutations.

### Dominant-negative effect of KCNQ2 mutations when co-expressed with KCNQ3

**Fig. 2:**
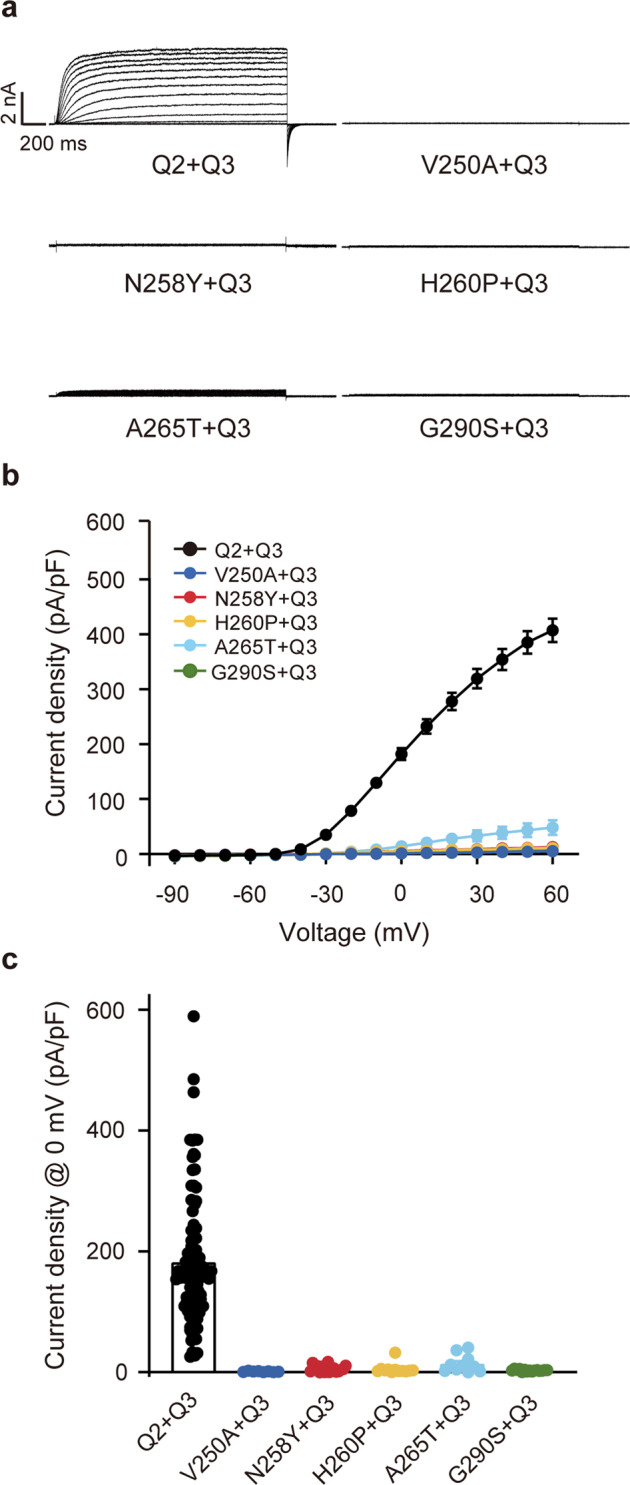
The pore mutations strongly suppressed heteromeric KCNQ2/3 currents. **a** Representative current traces, **b** current density-voltage plots, and **c** current densities at 0 mV from CHO cells co-transfected KCNQ2-WT or mutant channels with KCNQ3.

KCNQ2 alpha subunits can co-assemble with KCNQ3 alpha subunits to form a functional heteromeric KCNQ channel, which conducts a much stronger potassium current [5]. To determine the potassium current in a heteromultimer system, we first co-transfected these mutants of KCNQ2 and KCNQ3 at the ratio of 1:1. Dramatically, none of these mutations induced a detectable current, except A265T, which conducted a voltage-activated current significantly smaller than WT KCNQ2/3 current (14.0 ± 4.6 pA/pF vs. 182.1 ± 10.6 pA/pF at 0 mV, *P* < 0.0001, one-way ANOVA) (Fig. 2), indicating that the LOF effects caused by these KCNQ2 mutations were retained even when co-expressed with KCNQ3.

**Fig. 3:**
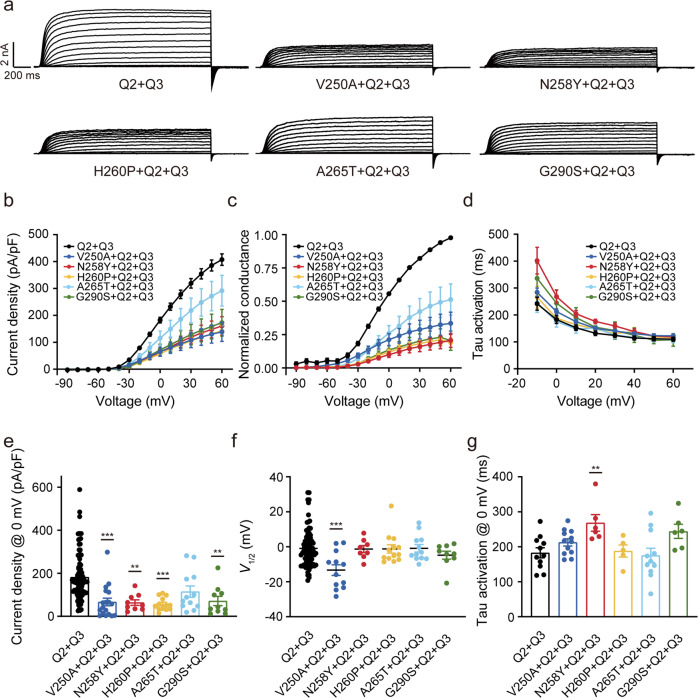
The pore mutations caused dominant-negative effects on the KCNQ2/3 heteromeric channels. **a** Representative current traces of the heteromeric channels. **b** Current density-voltage plots. **c** Conductance–voltage relationships generated by normalizing the tail current to the peak tail current of KCNQ2-WT. **d** Tau_activation_ from −10 mV to +60 mV. **e** Current densities at 0 mV, **f** *V*_1/2_, and **g** Tau_activation_ at 0 mV of heteromeric channels. Statistically significant differences are presented as: *P < 0.05, **P < 0.01, ***P < 0.001 versus Q2 + Q3.

Since patients carrying these KCNQ2 variants were all heterozygous, we co-transfected these variants with KCNQ2-WT and KCNQ3 cDNAs in a 1:1:2 ratio to mimic the heterozygous genotype. All the KCNQ2 mutant subunits co-expressed with KCNQ2-WT and KCNQ3 yielded much smaller currents than KCNQ2/KCNQ3 channel (Table 2, Fig. 3). While the current density of KCNQ2/3 was 182.1 ± 10.6 pA/pF at 0 mV, the current densities of V250A, N258Y, H260P, and G290S were suppressed by ~60%–70%, respectively to 66.8 ± 17.7 pA/pF (V250A, *P* < 0.0001, one-way ANOVA), 64.1 ± 12.9 pA/pF (N258Y, *P* = 0.0019, one-way ANOVA), 57.1 ± 8.3 pA/pF (H260P, *P* < 0.0001, one-way ANOVA), and 71.3 ± 21.5 pA/pF (G290S, *P* = 0.0023, one-way ANOVA); A265T suppressed the current density by 36%, to 114.9 ± 26.5 pA/pF (A265T, *P* = 0.09, one-way ANOVA) (Table 2, Fig. 3a, b, e), which was in accordance with the results when co-expressed with KCNQ3. Consistently, the channel conductances at all membrane potentials in mutants were dramatically lower than that in the WT channel (Fig. 3c). For the voltage-dependence, no differences were observed in *V*_1/2_ of most mutant heteromeric channels compared with WT KCNQ2/3 channel (−1.0 ± 1.0 mV), the *V*_1/2_ values of N258Y, H260P, A265T, and G290S were −1.3 ± 2.0 mV, −1.2 ± 2.4 mV, −0.9 ± 2.1 mV, and −4.8 ± 2.3 mV, respectively. Interestingly, the conductance-voltage (*G*-*V*) curve trended to a leftward shift in V250A, and the *V*_1/2_ value was left-shifted by ~12 mV (*V*_1/2_ = −13.4 ± 3.1, *P* = 0.0003, one-way ANOVA) compared with WT (Table 2, Fig. 3c, f). In addition, the activation time constants were also analyzed, and no change was observed in mutant heteromeric channels compared to the WT KCNQ2/3 channel except N258Y, which displayed a increased time constant at 0 mV (Table 2, Fig. 3d, g). These data suggested a strong dominant-negative effect of the five mutations.

**Table 2:** Current density and activation gating parameters of KCNQ2-WT and mutant channels.

Mutation	Current density@0 mV (pA/pF)	*V*_1/2_ (mV)	Tau@0 mV (ms)	RTG (10 μM)		XEN1101 (1 μM)		HN37 (1 μM)	
				*I*/*I*_0_	Δ *V*_1/2_ (mV)	*I*/*I*_0_	Δ *V*_1/2_ (mV)	*I*/*I*_0_	Δ *V*_1/2_ (mV)
KCNQ2	51.6 ± 3.7 (*n* = 90)	−7.1 ± 0.8 (*n* = 71)	232.0 ± 26.7 (*n* = 10)	1.3 ± 0.1 (*n* = 9)	-	1.7 ± 0.1 (*n* = 7)	-	3.1 ± 0.3 (*n* = 5)	-
V250A	0.5 ± 0.2 (*n* = 11)***	-	-	-	-	-	-	-	-
N258Y	0.4 ± 0.4 (*n* = 6)***	-	-	-	-	-	-	-	-
H260P	1.8 ± 0.7 (*n* = 10)***	-	-	-	-	-	-	-	-
A265T	1.6 ± 0.3 (*n* = 14)***	-	-	-	-	-	-	-	-
G290S	0.9 ± 0.4 (*n* = 12)***	-	-	-	-	-	-	-	-
KCNQ2 + KCNQ3	182.1 ± 10.6 (*n* = 100)	−1.0 ± 1.0 (*n* = 93)	182.7 ± 14.4 (*n* = 11)	2.0 ± 0.2 (*n* = 5)	−35.2 ± 1.4 (*n* = 5)	2.4 ± 0.4 (*n* = 5)	−41.1 ± 5.0 (*n* = 5)	4.0 ± 0.8 (*n* = 5)	−34.6 ± 6.3 (*n* = 5)
V250A + KCNQ3	1.1 ± 0.3 (*n* = 8)^###^	-	-	-	-	-	-	-	-
N258Y + KCNQ3	5.4 ± 1.3 (*n* = 17)^###^	-	-	-	-	-	-	-	-
H260P + KCNQ3	5.2 ± 2.5 (*n* = 12)^###^	-	-	-	-	-	-	-	-
A265T + KCNQ3	14 ± 4.6 (*n* = 10)^###^	−6.8 ± 7.0 (*n* = 3)^ns^	-	1.5 ± 0.1 (*n* = 7)	-	2.7 ± 0.3 (*n* = 5)	-	3.9 ± 0.4 (*n* = 5)	-
G290S + KCNQ3	2.9 ± 0.4 (*n* = 15)^###^	-	-	-	-	-	-	-	-
V250A+KCNQ2 + KCNQ3	66.8 ± 17.7 (*n* = 18)^###^	−13.4 ± 3.1 (*n* = 12)^###^	212.4 ± 10.2 (*n* = 12)ns	1.5 ± 0.1 (*n* = 5)	−30.0 ± 2.0 (*n* = 3)	1.9 ± 0.2 (*n* = 7)	−31.9 ± 7.0 (*n* = 3)	2.8 ± 0.3 (*n* = 5)	−22.9 ± 6.8 (*n* = 4)
N258Y+KCNQ2 + KCNQ3	64.1 ± 12.9 (*n* = 9)^##^	−1.3 ± 2.0 (*n* = 8)^ns^	268.1 ± 23.8 (*n* = 6)^##^	1.5 ± 0.1 (*n* = 5)	−37.8 ± 3.9 (*n* = 3)	2.0 ± 0.1 (*n* = 6)	−39.0 ± 6.6 (*n* = 5)	2.2 ± 0.2 (*n* = 5)	−37.5 ± 6.7 (*n* = 4)
H260P+KCNQ2 + KCNQ3	57.1 ± 8.3 (*n* = 13)^###^	−1.2 ± 2.4 (*n* = 13)^ns^	187.7 ± 17.4 (*n* = 5)^ns^	1.7 ± 0.1 (*n* = 5)	−32.2 ± 0.9 (*n* = 3)	2.0 ± 0.1 (*n* = 6)	−34.0 ± 1.5 (*n* = 5)	2.1 ± 0.2 (*n* = 7)	−37.3 ± 5.5 (*n* = 4)
A265T+KCNQ2 + KCNQ3	114.9 ± 26.5 (*n* = 12)^ns^	−0.9 ± 2.1 (*n* = 12)^ns^	175.2 ± 20.5 (*n* = 11)^ns^	1.6 ± 0.1 (*n* = 6)	−36.9 ± 1.6 (*n* = 5)	2.3 ± 0.2 (*n* = 7)	−38.1 ± 5.0 (*n* = 5)	2.5 ± 0.3 (*n* = 6)	−36.4 ± 2.2 (*n* = 4)
G290S+KCNQ2 + KCNQ3	71.3 ± 21.5 (*n* = 10)^##^	−4.8 ± 2.3 (*n* = 9)^ns^	243.9 ± 20.2 (*n* = 6)^ns^	1.4 ± 0.1 (*n* = 6)	−30.9 ± 5.0 (*n* = 3)	1.8 ± 0.1 (*n* = 6)	−25.9 ± 5.2 (*n* = 3)	2.4 ± 0.2 (*n* = 9)	−32.7 ± 3.7 (*n* = 4)

*n* Number of cells recorded.

***P < 0.001 versus KCNQ2; ^##^*P* < 0.01, ^###^*P* < 0.001 versus KCNQ2/3.

### Cell-surface protein expression levels of the pore mutations was normal

**Fig. 4:**
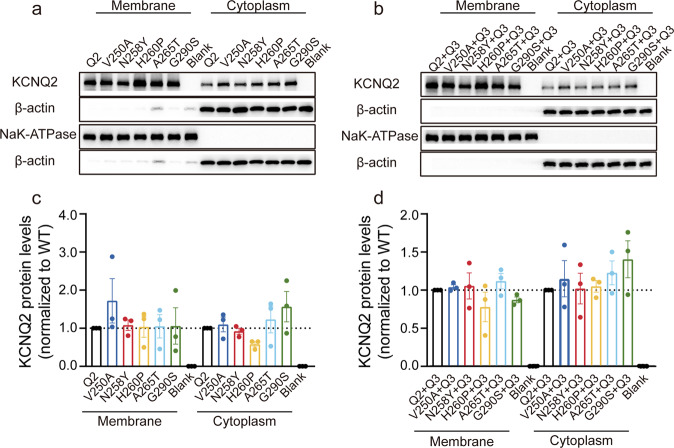
Membrane expression of WT and mutant KCNQ2 subunits was unaffected when transfected alone and co-transfected with KCNQ3 subunits. Western blot analysis of membrane proteins (left lanes) and cytoplasmic protein (right lanes) from CHO cells **a** expressing KCNQ2 variants alone and **b** co-expressing KCNQ2 with KCNQ3 subunits. Relative membrane expression levels of KCNQ2 subunits when **c** transfected alone and **d** co-transfected with KCNQ3 subunits.

We further evaluated whether the observed decrease in current was caused by changes in the expression level of channel proteins. The pathologic KCNQ2 variants were co-transfected alone or with KCNQ3 into CHO cells in a 1:1 ratio, and Western blotting studies were applied to analyze the cytosolic and membrane protein expression of KCNQ2. As shown in Fig. 4, the mutant and KCNQ2-WT subunits expressed in the membrane were more abundant than that in the cytoplasm; there was no significant difference in the expression level on the cell membrane between WT and mutants based on the quantitative results of the Western blotting.

### KCNQ openers rescued subthreshold current density in heterozygous channels

**Fig. 5:**
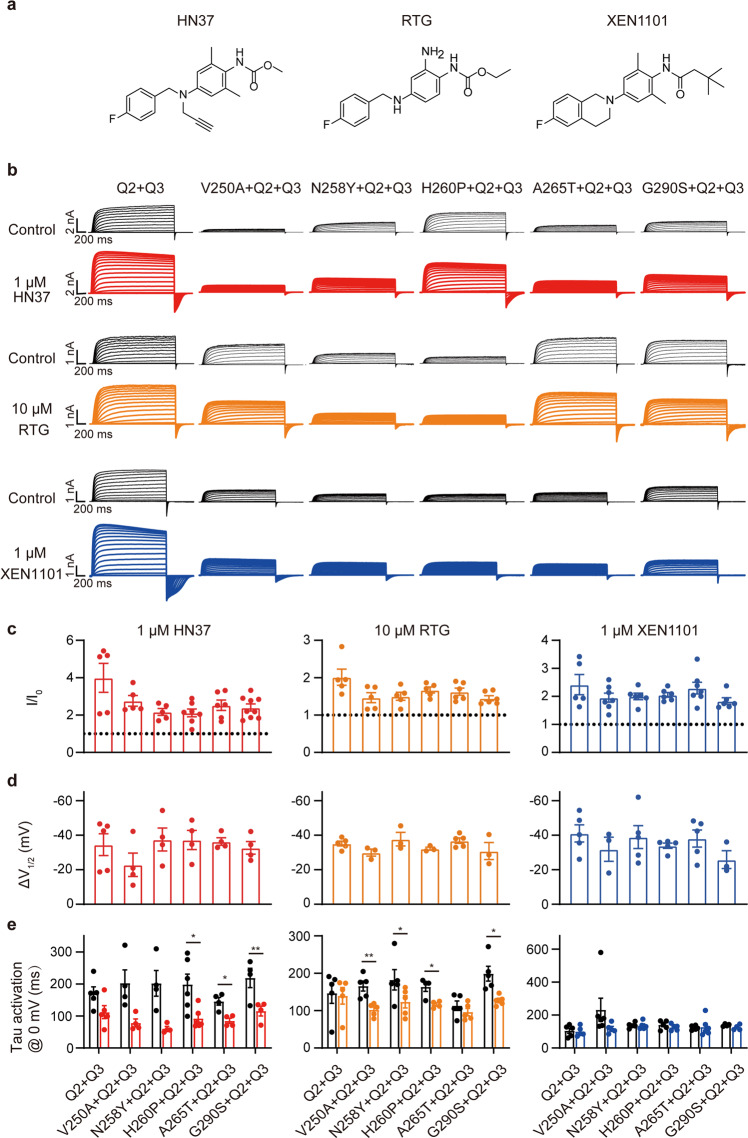
The LOF effects of KCNQ2 mutations were rescued by KCNQ openers when co-transfected with KCNQ2-WT and KCNQ3. **a** Chemical structural formulas of HN37, RTG, and XEN1101. **b** Representative current traces of KCNQ2 mutant channels co-transfected with KCNQ2-WT and KCNQ3 before and after application of 1 μM HN37 (red), 10 μM RTG (orange), or 1 μM XEN1101 (blue). Effect of 1 μM HN37, 10 μM RTG, and 1 μM XEN1101 on the **c** *I*/*I*_0_ at 0 mV, **d** Δ*V*_1/2_, and **e** Tau_activation_ at 0 mV of heteromeric channels.

According to previous reports, some KCNQ2 LOF mutations could be rescued by KCNQ openers in the homomeric configuration [8, 29–31]. However, the five mutations in this study did not conduct KCNQ current even after applying KCNQ openers, including RTG and its analogs (HN37, and XEN1101) and ICA-069673, which bind to a different binding site from RTG [32–34] (Supplementary Figs. S2–S5). Since most patients with KCNQ2 encephalopathy carry a heterozygous mutation, we evaluated the ability of KCNQ openers in clinical trials (RTG, HN37, and XEN1101) to rescue the function of mutations in the heteromeric configuration. RTG was used at a concentration of 10 μM (near the EC_70_ concentration), while the two candidates subjected to clinical trials, XEN1101 and HN37, were used at a concentration of 1 μM based on their >10-fold improved potency compared to RTG in the previous results [31, 33]. The KCNQ2/3 currents were significantly increased when the cells were exposed to RTG (10 μM), HN37 (1 μM), or XEN1101 (1 μM) (Table 2, Supplementary Figs. S2–S4). However, no significant current could be detected in the cells expressing the four KCNQ2 mutations with KCNQ3 in a ratio of 1:1 when exposed to KCNQ openers at the same concentration, except A265T, of which the current when co-expressed with KCNQ3 increased about 1.5-, 3.9-, and 2.7-folds by applying RTG, HN37, and XEN1101, respectively (Table 2, Supplementary Figs. S2–S4). When KCNQ openers were applied to the five variants co-expressed with WT KCNQ2, and KCNQ3 cDNAs in a 1:1:2 ratio, the heteromeric currents at 0 mV of the five mutations were significantly increased, the *V*_1/2_ of the five mutants was shifted leftward significantly, and the activation time constant values (Tau_activation_) were significantly decreased at membrane potentials of −10 to 60 mV (Table 2, Fig. 5). These data demonstrated that the KCNQ openers efficiently rescued the impaired KCNQ channel in the physiological heterozygous configuration.

## Discussion

This work described the clinical features, electrophysiological function, and membrane expression in five KCNQ2 pore mutations causing DEE. We found that all these mutations lead to nonconducting channels when transfected alone; LOF effects were retained even in heteromeric channels when co-transfected with KCNQ3 or with KCNQ2 and KCNQ3 subunits. In addition, we evaluated the pharmacological sensitivity of these mutated channels to KCNQ openers, including an approved antiepileptic drug RTG and two candidates subjected to clinical trials HN37 and XEN1101. We found that the heteromeric mutant currents, when co-expressed with the WT KCNQ2 and KCNQ3 at a ratio of 1:1:2, could be rescued by KCNQ openers, suggesting affected children might get potential benefit from KCNQ openers for both seizure control and development rescue.

### New and previously reported KCNQ-DEE hotspots in the pore-forming domain

The mutations in this work affected five positions at the pore-forming region of KCNQ2, while three of them, V250A, A265T, and G290S, have currently been uploaded to the ClinVar database (https://www.ncbi.nlm.nih.gov/clinvar/) without functional evaluation [12, 13, 35], the two mutations, H260P, and N258Y are reported for the first time in this study. The A265 residue has been reported to change to V, P, and T in the database or previous work, and the channel function of A265P has been evaluated before [8, 35]; the A265P failed to conduct potassium current without affecting the membrane expression when expressed alone, and the co-expression partially rescued the impairment with KCNQ3, similar to the electrophysiological abnormalities of A265T in this study, and this mild functional impairment might be related to the milder phenotypes of the two patients (patients 5 and 6). The G290 residue has been reported to change to V, D, and S in the database or previous work [13, 35], and the channel function of G290D has been assessed previously [8]; the G290D failed to conduct potassium current when expressed alone, and this impairment was retained even when co-expressed with KCNQ3, consistent with the electrophysiological abnormalities of G290D in this study. The N258 residue has been reported to change to K and S in the database or previous work, and the channel function of N258S has been estimated earlier [30]; the N258S only conducted a small potassium current, and the membrane expression was dramatically reduced when expressed alone, and the channel function was partially rescued by the co-expression with KCNQ3, different from the electrophysiologic abnormalities of N258Y reported here. In addition, the V250 residue has been reported to change to V, P, and T in the database. The various substitutions of the same residue make these residues hotspots for KCNQ2-DEE. Interestingly, H260 was the only non-conserved one among the five residues, and the corresponding residue in other KCNQ family members was a negatively charged E or D, suggesting the residue charge at this position was not important for maintaining the conformation of the KCNQ selectivity filter; in ClinVar database, a change to a Y was reported to be associated with KCNQ2-DEE, and the change from the H to a P or Y might impair the structure around the GYG, even though the residue was not conserved.

### KCNQ2-DEE mutations located in the pore domain usually induce a loss of function

KCNQ2 epilepsy-causing variants can be classified according to diverse parameters, including location, function, and related clinical phenotypes. Generally, it is considered that missense mutations found in KCNQ2-related epilepsy cases were mainly located in several hotspots, including the S4 voltage sensor, the pore, and the calmodulin-binding helix. According to the channel function, the missense mutation can be divided into LOF or gain of function (GOF), and the LOF effects can be further divided into haploinsufficiency or dominant-negative effects. Furthermore, the correlations between the genotypes and the phenotypes in the KCNQ channel have been focused on for a long time. Generally, the affected characteristics of these mutations can be predicted by their location. For example, pathogenic mutations in KCNQ2 VSD typically affect the voltage dependence of activation; while the majority, including R207W and R213W, causes a depolarizing shift of the *G*-*V* curve, the minority fraction induces a GOF and allows the channels to be more easily opened by voltage, and almost all known pathogenic KCNQ2 GOF mutations are located in the VSD [36–38]. Epilepsy-causing mutations were also densely distributed in the pore-forming domain of KCNQ2, and these mutations always lead to a LOF by impairing transmembrane potassium conduction [8, 21, 39]; a recent work by Wu et al. [40] revealed a similar distribution pattern in deafness-related KCNQ4 mutations, characterizing the function of KCNQ4 missense mutations on a large scale. In consistence with this point, all five pore mutations in this study affected the current density of the KCNQ2 channel in both homomeric and heteromeric patterns, and most of them did not influence the voltage dependence except V250, which is located in the S5 that accepted the conformational changes from the voltage-sensing S4.

### Application of KCNQ openers as a precision therapy in KCNQ2-related epilepsy

Genetic diagnosis and electrophysiological function analysis facilitate the understanding of genotype-phenotype correlation in KCNQ2-DEE and contribute to the implementation of precision therapy in KCNQ2-DEE using KCNQ openers. Retigabine, the first marketed KCNQ opener as anti-seizure medication (ASM), was well tolerated and potentially beneficial against KCNQ2-DEE when started early [23, 41], consistent with the fact that certain variants with LOF mutation can be fixed using KCNQ channel agonists in vitro [29, 42]. Thus, although RTG has been withdrawn due to side effects like skin discoloration, its proprietary pediatric formulation has received Orphan Drug Designation for treating seizures associated with KCNQ2-DEE from the FDA because of the irreplaceable therapeutic effects of KCNQ channels opener (XEN496, trial number. NCT04639310 and NCT04912856). However, multiple cases of neonatal epilepsy due to KCNQ2 GOF mutation have also been reported so far, and these patients cannot benefit from KCNQ opener, which may exacerbate their epileptic phenotypes [23]. In addition, when KCNQ2 mutations are coincidentally located in the determinants of KCNQ opener action, or when mutations cause a total loss of function of the potassium current, the effects of KCNQ opener would be attenuated or even abolished, suggesting the limitation of KCNQ opener usage in the treatment of KCNQ2-related epilepsy [43, 44]. Fortunately, KCNQ2 encoding K_V_7.2 subunit heterodimerizes with K_V_7.3 in the physiological condition, and the heteromeric K_V_7.2/3 channel conducts a potassium current ~10 times larger than the K_V_7.2 homomer. Moreover, most patients with KCNQ2 encephalopathy carry a heterozygous mutation; thus, the pharmacological responses in the condition when mutation co-expressed with KCNQ2 WT and KCNQ3 (1:1:2 ratio) may better represent the therapy response. In this study, although the five mutations did not conduct KCNQ current even after applying KCNQ openers, the mutant currents were significantly activated by RTG, HN37, or XEN1101 when co-expressed with KCNQ2 WT and KCNQ3 in a ratio of 1:1:2, regardless of the non-conductance when expressed alone. These results suggest that the KCNQ2 encephalopathy patients with LOF mutation might potentially benefit from KCNQ opener, consistent with previous work by Nissenkorn et al. [41], in which a patient carrying a LOF benefited from the application of RTG.

In conclusion, our study described the clinical characteristics, electrophysiological phenotypes, and response to KCNQ openers in vitro of five newly identified KCNQ2 pore mutations from patients diagnosed with KCNQ2-DEE. These pore mutations caused LOF effects, and the current impairment was retained even when co-expressed with WT KCNQ2 and KCNQ3, which could be rescued by the KCNQ openers.

## Supplementary information

Supplementary Figure S1

Supplementary Figure S2

Supplementary Figure S3

Supplementary Figure S4

Supplementary Figure S5

Supplementary Information
