# Supplementary figures and images for: Functional characterization and in vitro pharmacological rescue of KCNQ2 pore mutations associated with epileptic encephalopathy
Source: Acta Pharmacol Sin. 2023 Mar 17;44(8):1589–99. doi: 10.1038/s41401-023-01073-y (PMC10374643; doi:10.1038/s41401-023-01073-y)

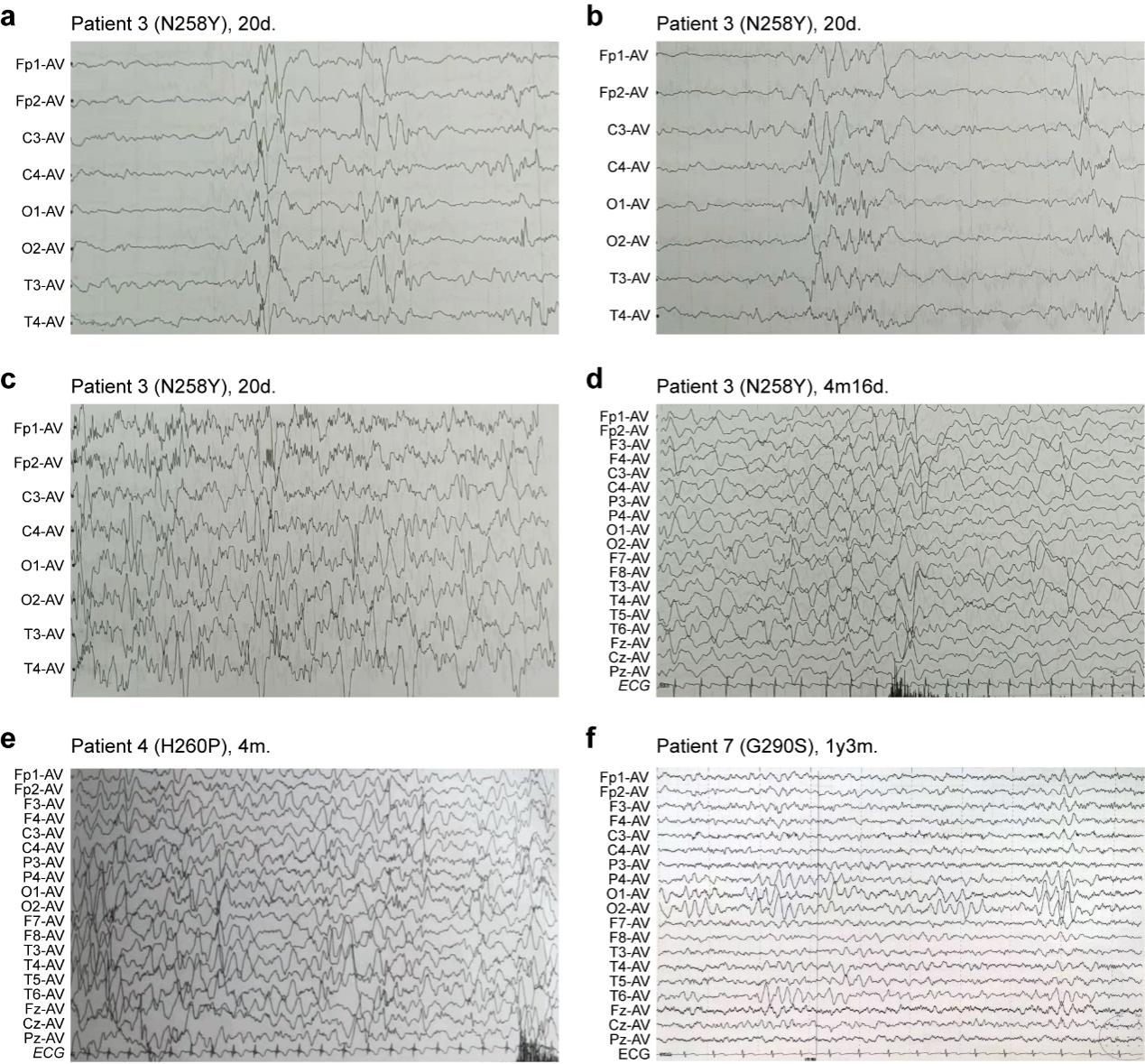

Supplement: Supplementary file 1 — Supplementary Figure S1 [file 41401_2023_1073_MOESM1_ESM.tif]

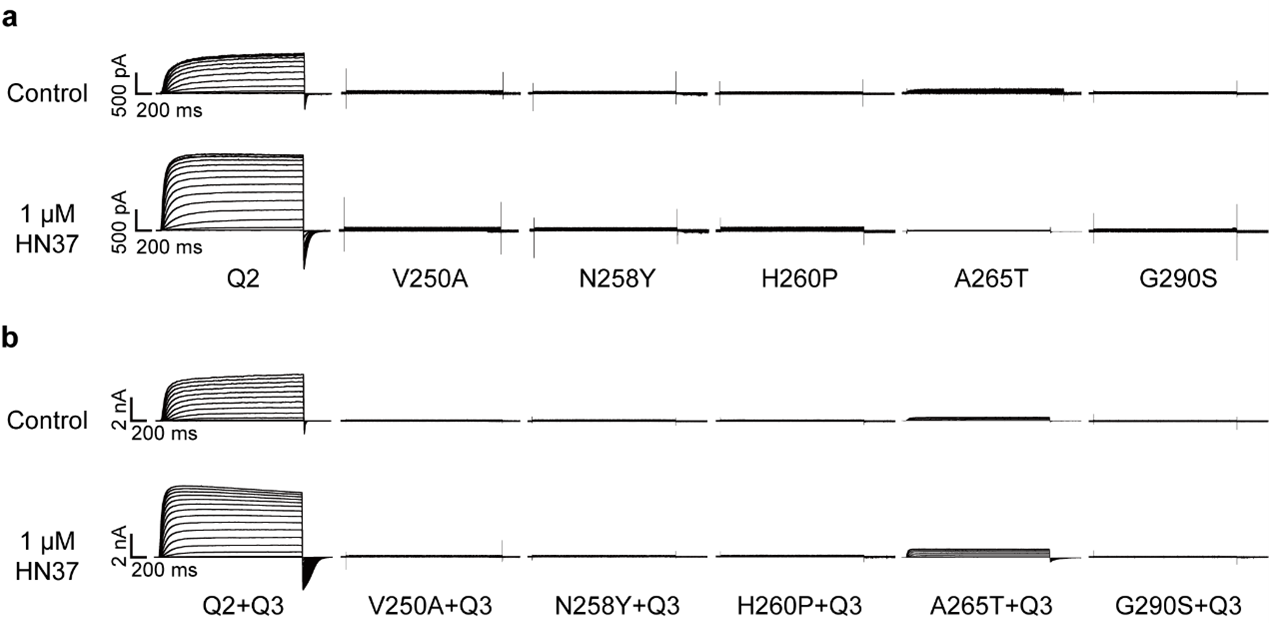

Supplement: Supplementary file 2 — Supplementary Figure S2 [file 41401_2023_1073_MOESM2_ESM.tif]

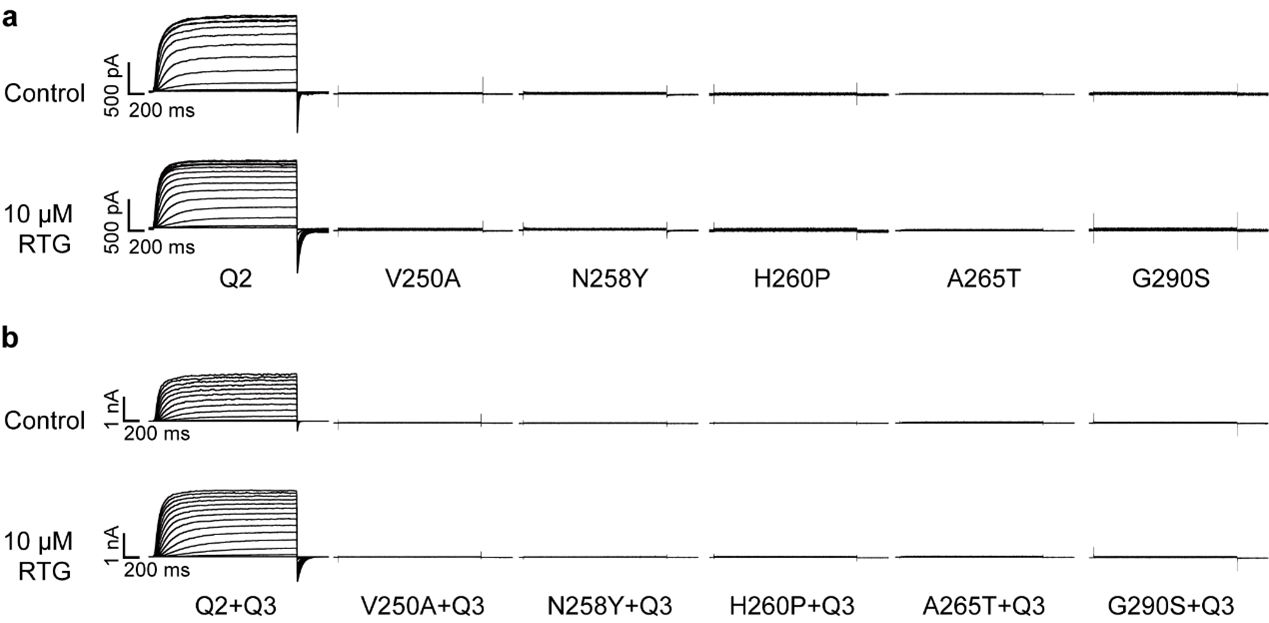

Supplement: Supplementary file 3 — Supplementary Figure S3 [file 41401_2023_1073_MOESM3_ESM.tif]

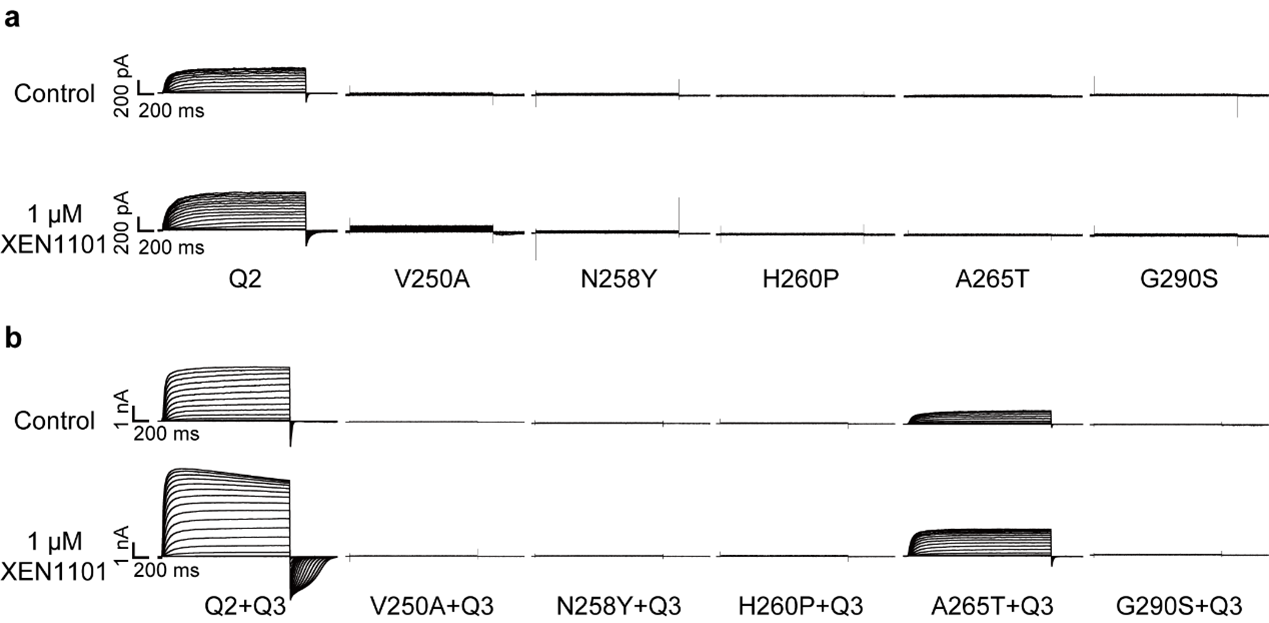

Supplement: Supplementary file 4 — Supplementary Figure S4 [file 41401_2023_1073_MOESM4_ESM.tif]

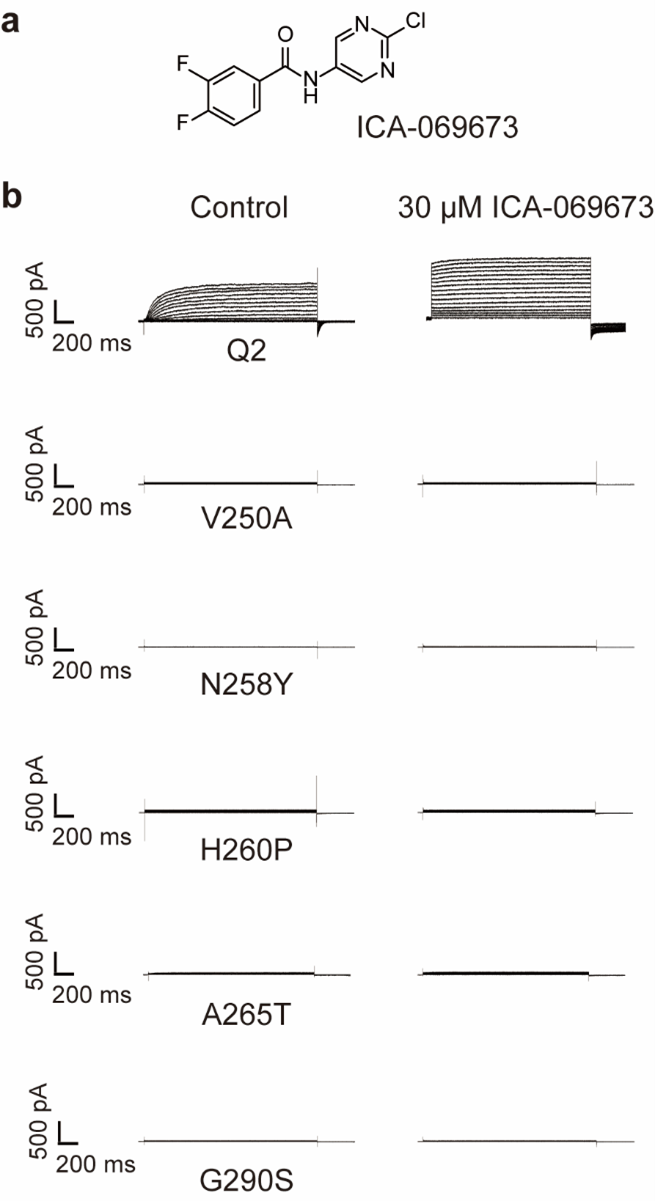

Supplement: Supplementary file 5 — Supplementary Figure S5 [file 41401_2023_1073_MOESM5_ESM.tif]
