# Supplementary material for: Functional characterization and in vitro pharmacological rescue of KCNQ2 pore mutations associated with epileptic encephalopathy
Source: Acta Pharmacol Sin. 2023 Mar 17;44(8):1589–99. doi: 10.1038/s41401-023-01073-y (PMC10374643; doi:10.1038/s41401-023-01073-y)
Supplement: Supplementary file 6 — Supplementary Information [file 41401_2023_1073_MOESM6_ESM.docx]

**Supplementary information**

**Fig. S1 Representative EEG of KCNQ2-related encephalopathy patients. a b** The burst-suppression pattern EEG within the 1^st^ month (patient 3, 20 day). **c** Multifocal epileptic activity with many sharp-slow and spike-slow complex in the EEG during clinical seizures (patient 3, 20 day). Atypical or discontinuous hypsarrhythmia in the EEG around 3-5 months from **d** patient 3 (4 month 16 day) and **e** patient 4 (4 month). **f** Slow background of EEG in accordance to age, delayed rhythm of occipal lobes was shown (patient 7, 1 year 3 month).

**Fig. S2 Effect of 1 μM HN37 on KCNQ2 and KCNQ2/KCNQ3 channels. a** Representative current traces of KCNQ2 channel before and after 1 μM HN37. **b** Representative current traces of KCNQ2/3 channel before and after 1 μM HN37.

**Fig. S3 Effect of 10 μM RTG on KCNQ2 and KCNQ2/KCNQ3 channels. a** Representative current traces of KCNQ2 channel before and after 10 μM RTG. **b** Representative current traces of KCNQ2/3 channel before and after 10 μM RTG.

**Fig. S4 Effect of 1 μM XEN1101 on KCNQ2 and KCNQ2/KCNQ3 channels. a** Representative current traces of KCNQ2 channel before and after 1 μM XEN1101. **b** Representative current traces of KCNQ2/3 channel before and after 1 μM XEN1101.

**Fig. S5 Effect of 30 μM ICA-069673 on KCNQ2 channels. a** Chemical structural formula of ICA-069673. **b** Representative current traces of KCNQ2 channel before and after 30 μM ICA-069673.
